# Prey Vulnerability Limits Top-Down Control and Alters Reciprocal Feedbacks in a Subsidized Model Food Web

**DOI:** 10.1371/journal.pone.0085830

**Published:** 2014-01-21

**Authors:** William I. Atlas, Wendy J. Palen

**Affiliations:** Earth to Ocean Research Group, Department of Biological Sciences, Simon Fraser University, Burnaby, Canada; Dauphin Island Sea Lab, United States of America

## Abstract

Resource subsidies increase the productivity of recipient food webs and can affect ecosystem dynamics. Subsidies of prey often support elevated predator biomass which may intensify top-down control and reduce the flow of reciprocal subsidies into adjacent ecosystems. However, top-down control in subsidized food webs may be limited if primary consumers posses morphological or behavioral traits that limit vulnerability to predation. In forested streams, terrestrial prey support high predator biomass creating the potential for strong top-down control, however armored primary consumers often dominate the invertebrate assemblage. Using empirically based simulation models, we tested the response of stream food webs to variations in subsidy magnitude, prey vulnerability, and the presence of two top predators. While terrestrial prey inputs increased predator biomass (+12%), the presence of armored primary consumers inhibited top-down control, and diverted most aquatic energy (∼75%) into the riparian forest through aquatic insect emergence. Food webs without armored invertebrates experienced strong trophic cascades, resulting in higher algal (∼50%) and detrital (∼1600%) biomass, and reduced insect emergence (−90%). These results suggest prey vulnerability can mediate food web responses to subsidies, and that top-down control can be arrested even when predator-invulnerable consumers are uncommon (20%) regardless of the level of subsidy.

## Introduction

Over the last three decades ecologists have increasingly recognized the importance and ubiquity of resource subsidies linking adjacent food webs [1]–[3]. These flows which can include organisms, material, and nutrients from outside local food webs can fundamentally alter the dynamics of ecosystems [2], increasing the productivity of recipient ecosystems, [2], [4] and altering the strength of interactions among species in recipient communities [2], [5]–[7]. Consequently, consumer biomass in subsidized ecosystems often exceeds that which can be sustained by *in situ* production alone [8], [9]. In such instances, access to resource subsidies decouples predator population dynamics from local resources, and communities can experience strong top-down control [10], [11] and strengthened trophic cascades [2], [12], [13]. However, the composition of prey and predator assemblages may influence the strength of interactions between trophic levels in recipient food webs [14]. In cases where behavioral or morphological traits among prey limit their vulnerability to predation, the flow of energy to higher trophic levels can be arrested, weakening top-down control over primary consumer biomass [15]–[21]. While resource subsidies have been investigated across a range of ecosystems, the degree to which prey vulnerability mediates food web responses to resource subsidies remains poorly understood.

Tributary stream ecosystems receive large inputs of both detritus and prey from the surrounding terrestrial environment, and the importance of resource subsidies for stream food webs is well documented [1], [3], [22]. In small tributaries, where dense canopy cover limits in situ production, terrestrial detritus and leaf litter contribute disproportionately as a source of energy at the base of the food web [1]. Direct inputs of terrestrial prey are also an important source of energy for stream predators, and falling terrestrial invertebrates can represent more than 50% of predatory fish diets [3]. Aquatic invertebrates that emerge into the surrounding forest as adults provide important prey for many riparian predators [9], [11].

Theoretical work suggests that highly subsidized stream ecosystems should experience strong top-down control [13]. However, experimental evidence to support this theory is limited, and in some cases terrestrial subsidies may reduce predation intensity on aquatic prey [5], [7]. The degree to which prey subsidies strengthen top-down control in stream food webs should depend on the degree to which prey subsidies support the biomass of predators, and the ability of subsidized predators to exploit local prey. Armored caddisflies, which build cases from rocks, sand, and organic material are often abundant in temperate stream food webs, and are largely invulnerable to predation by fish and amphibians [17], [20]. Wootton, Parker & Power [15] demonstrated that the presence of these large armored grazers can inhibit trophic cascades in the Eel River, California. However, the mainstem Eel River food web is autotrophic, and the degree to which patterns of top-down control are influenced by the presence of armored primary consumers may differ in highly subsidized tributary streams. The role of prey invulnerability in mediating the strength of top-down control in subsidized food webs has not been explicitly studied, and we sought to understand the degree to which food web responses to resource subsidies are limited by the presence of armored prey.

Here we use a multi-trophic model as used by e.g. [15], [23] parameterized from a large field experiment and extensive surveys to test the response of tributary stream food webs to variation in the magnitude of terrestrial prey subsidies, and the degree to which the presence of armored invertebrates limits the propagation of subsidy effects through the food web. Short-term dynamics were modeled for two top predators, steelhead trout (*Oncorhychus mykiss*) and Pacific giant salamander (*Dicaptodon tenebrosus*), armored and vulnerable aquatic invertebrates partitioned into two general guilds based on their feeding ecology (herbivore, detritivore), as well as algal and detrital resource pools. To test the degree to which the presence of armored invertebrates altered the strength of top-down control by each predator species, food web responses were simulated across a range of invertebrate community assemblage scenarios ranging from 0% to 100% armored taxa by biomass. Changes in the biomass of predators, herbivores, detritivores, the biomass of emerging aquatic invertebrates, and basal energy pools (algae, detritus) were also modeled across a range of prey subsidy inputs (0 to 200% observed natural flux). The aim of this research was to test the prediction that terrestrial prey subsidies would increase the biomass of both Pacific giant salamanders and steelhead trout, and that increased predator biomass would strengthen top-down control, reducing the biomass of aquatic invertebrates and the magnitude of the reciprocal subsidy into the riparian forest via emergence. In contrast, if the strength of top-down control by subsidized predators within stream food webs was limited by the composition of the prey community, then the strength of cascading trophic interactions may decline rapidly as the proportion of armored invertebrates in the benthic community increased. If top-down control within the model food webs is diminished by the presence of armored prey, we predicted that changes in subsidy magnitude would play a relatively small role in determining primary consumer and primary producer biomass.

## Methods

### Ethics Statement

This work was conducted under California Department of Fish and Game (#11077), NOAA (#14904), and Simon Fraser University Animal Care (920B-09) permits. Steelhead in Fox creek are protected as “Threatened” under the US Endangered Species Act, however all sampling for this study was permitted by NOAA under a 4(d) permit allowing sampling of threatened species.

### Model Overview

The dynamics of tributary stream food webs were modeled using a multi-trophic modeling framework [15], [23], written and executed as a discrete model in visual basic and excel. The model included a series of linked equations including two top predators (Pacific giant salamander and steelhead trout), both armored and vulnerable primary consumers (herbivores and detritivores), algae, and detritus. Model parameters were taken either from empirical values measured in Fox Creek, a tributary of the South Fork Eel River, or from the literature. Resources entered the model food web through three pathways, *in situ* primary production of algae, inputs of terrestrial detritus, and inputs of terrestrial invertebrate prey. Both terrestrial detritus and terrestrial prey were modeled as donor controlled subsidies [2] that entered the food web at a constant rate estimated in the field and varied in subsequent simulations. Using this multi-trophic modeling framework changes in the size of each biomass pool were related to a range of model scenarios. For the purposes of studying stream food webs, the high species diversity of stream invertebrates is often simplified into functional feeding groups (e.g. shredders, scrapers, collectors, predators), and these functional groups may derive their biomass from both aquatic and terrestrial energy sources [24]. However, because we sought to test effects of subsidized predation across all functional groups, biomass and diversity of stream invertebrates were simplified into two pools (algivores and detritivores) relying exclusively on each of the two primary energy sources for stream secondary production. Invertebrate biomass was further categorized into two groups based on their vulnerability to predation as observed from survey data and previous diet studies [25], [26], with one group vulnerable to both predator species and another group with physical armoring that was only marginally vulnerable to salamanders and were entirely invulnerable to predatory steelhead.

### Algae and Detritus

Changes in the biomass of algae (A) were modeled (Equation 1) as a function of the availability of light and consumption by grazers (c_a_) [15]. The conversion efficiency of light to algae was set at b_a_ = 0.15, since transfer efficiencies for photosynthic rates relative to canopy cover are not available, we chose a biologically plausible value that supported levels of primary productivity similar to those measured in Fox creek [20]. The standing biomass of algae (A = 1.59±0.53 g·m^−2^) was measured empirically, light availability expressed as average % canopy cover (L = 90±6%) from empirical values measured in Fox creek, and a theoretical consumption rate of algae by grazers (c_a_) from the literature [27]. Algal biomass in the model system was limited by grazing as a function of the consumption rate of algivores (c_alg_) which included both vulnerable (H_alg_) and armored (G_alg_) members. Background loss rates were not included as consumption rates were relatively high and on the timescales we modeled, background loss did not appreciably affect algal biomass.
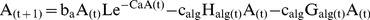
(1)


Inputs of detritus were constant (I) and detritus biomass declined due to consumption by armored (G_det_) and vulnerable (H_det_) detritivores (Equation 2). Consumption by detritivores was a product of the daily consumption rate (c_det_), the biomass of detritivores, and the standing stock of detritus (D).
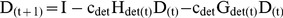
(2)


### Aquatic Invertebrates

Vulnerable aquatic invertebrate biomass (H) was modeled separately for algivores (H_alg_) (Equation 3) and detritivores (H_det_) (Equation 4). Changes in invertebrate biomass were estimated as a function of their daily consumption rate of basal energy, which differed for algivores (c_alg_) and detritivores (c_det_), their growth efficiency (b_alg_ and b_det_), and four sources of biomass loss: predation by trout and salamanders, emergence (e_h_) into aerial adults, and background natural mortality (m_h_). Consumption rates for detritivores (c_det_) were estimated at 0.35 g·g^−1^·day^−1^ [28], [29], and consumption rates for algivores (c_alg_) were estimated at 0.2 g·g^−1^·day^−1^ [28]. Growth efficiencies for detritivores (b_det_) and algivores (b_alg_) were set at 0.065 g·g^−1^ and 0.195 g·g^−1^ respectively [30], reflecting the relatively poor nutritional quality of most detrital food. Predation by both trout and salamanders was modeled using a type III functional response that limited exploitation of prey at low densities and saturated at high prey densities [31]. Both trout and salamanders exhibit wide diet breadth in Fox creek [25] [W. Palen unpublished data] and the type III functional response allowed reduced predation intensity at low abundance and saturation at high abundance based on maximum observed prey intake. Laboratory feeding trials have shown that as prey abundance increases fish feeding rates typically saturate, lending support for the use of a type II functional response [32], however these experiments are often conducted in highly simplified environments. In choosing a type III functional response for our two predator species we hoped to capture the effects of habitat complexity on predation rates [33], and importance of prey body size for predation rates [32], [34], such that even during periods of high predation intensity smaller more cryptic vulnerable invertebrates were able to persist. Consumption rates by predators were a function of each predator’s daily consumption (c_t_ and c_s_), the total vulnerable prey density (H), and half saturation constant for predators, set based on observed maximum diet size in Fox creek (α). Total predation on each functional group (herbivore, detritivores) of vulnerable aquatic invertebrates was therefore a product of the predator specific consumption rates, the biomass of the prey pool and the biomass of the two predators (T, S).
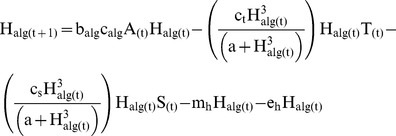
(3)

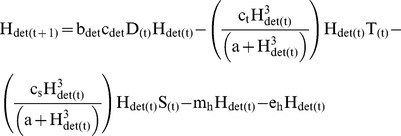
(4)


The biomass of armored invertebrates changed as a function of consumption of algae (c_alg_) or detritus (c_det_) and the growth efficiency of each functional group (b_alg_ and b_det_), and was limited by salamander predation and two sources of non-predator loss (emergence and background mortality) as above (Equations 5 & 6). Unlike vulnerable invertebrates, armored taxa in our model were not subject to predation by trout and experienced only limited predation by salamanders. Diet data from Fox Ck. indicates that armored invertebrates are almost never consumed by trout, even when terrestrial prey subsidies were experimentally reduced [35]. Diet studies of Pacific giant salamander found that armored taxa comprised only 6.5% of salamander diets by volume even when armored invertebrates were the majority of invertebrate biomass in a tributary stream [25]. As such, we modeled predation by salamanders on armored taxa with the type III functional response but with total consumption limited to a maximum of 6.5% of their daily consumption rate (c_s_).
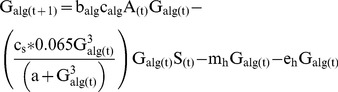
(5)

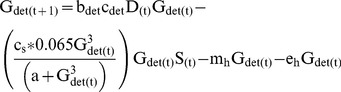
(6)


The emergence coefficient (e_h = _0.039) was determined based on an empirically derived linear relationship between the total biomass of benthic invertebrates and the biomass of total daily aquatic invertebrate emergence from a large field experiment [35]. The biomass of emerging aquatic invertebrates was estimated from the biomass of vulnerable (H) and armored (G) groups (Equation 7).
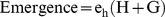
(7)


### Predators

Changes in steelhead trout (*O. mykiss*), and Pacific giant salamander (*D. tenebrosus*) biomass were modeled separately because of differences in their resource use. While the two species do overlap in their consumption of many types of aquatic invertebrate prey [25], [36], steelhead trout in tributary stream food webs are known to rely heavily on terrestrial invertebrates for their growth [3]. Terrestrial prey constitute a much smaller fraction of the diets of salamanders than aquatic invertebrate prey [25]. Growth efficiency was set to 10% for both predators, based on the assumption that it was similar and constant for the two vertebrate predators [37].

Trout biomass changed as a function of their growth efficiency (b_t_), consumption of vulnerable aquatic invertebrates biomass (H), and consumption of terrestrial prey (X) (Equation 8). Consumption of vulnerable aquatic invertebrates by trout was modeled as a type III functional response [31]. Because terrestrial prey subsidies are donor controlled, trout consumption of this resource was modeled as the product of the daily consumption rate of trout (c_t_) and the biomass of trout (T). However, daily consumption of terrestrial prey subsidies by trout (c_t_T) could not exceed the total magnitude of the input (X). Consumption rates for predatory steelhead trout (c_t_) were estimated from a published temperature-dependent consumption relationship [38] using daily stream temperatures measured in Fox creek (see below) from early-July to late-August 2010 averaged across the summer (July 3rd–August 22nd) to produce a single rate (0.075 g·g^−1^·day^−1^).
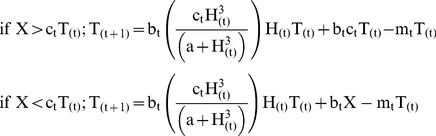
(8)


Salamander biomass was similarly modeled as increasing due to consumption of vulnerable aquatic invertebrates but also included limited consumption of armored invertebrates (Equation 9). Salamanders benefitted from terrestrial prey only when trout biomass was incapable of fully exploiting the resource (ie X>c_t_T). The pool of terrestrial prey available to salamanders was therefore modeled as (X - c_t_T). Like trout, salamanders’ maximum consumption of terrestrial prey c_s_S could not exceed the magnitude of the available prey subsidies, and the maximum potential salamander consumption of terrestrial prey could not exceed the size of the pool of available prey (X- c_t_T). Consumption rates of salamanders (c_s_) were estimated directly from diets of Pacific giant salamanders assuming a 6-hour gut clearance time (0.02236 g·g^−1^·day^−1^) [R.G. Munshaw, unpublished data].
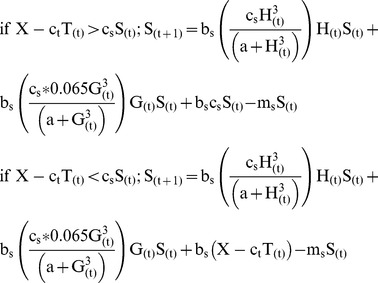
(9)


All consumption rates and growth efficiency parameters were held constant in the model.

### Model Starting Conditions

Starting biomass in each trophic level, subsidy flux rates for the reference model, and the relationship between emergence and standing benthic invertebrate biomass in the model were set from values measured in Fox creek, Mendocino Co., California (2.8 km^2^ drainage area), a tributary of the South Fork Eel River (39° 43′45′′ N, 123° 38′40′′ W). Fox creek supports low algal productivity due to dense canopy cover (average ∼ 90%), receives large inputs of terrestrial prey (0.261±0.051 g·m^−2^ ·day^−1^) [35], and supports populations of both Pacific giant salamanders and steelhead trout. Average percent canopy cover was estimated using spherical densitometer from a series of 32 pools. Starting biomass of steelhead trout and salamanders used were based on densities estimated from depletion sampling the same 32 pools (∼1 km) of the creek in late June 2010. Predators were surveyed using a combination of snorkel, hand capture, and electro-fishing until no new animals were captured. All animals were weighed (g), and pool areas measurements allowed estimates of biomass per unit area (steelhead = 5.12±3.03 g·m^−2^, salamanders = 13.42±6.85 g·m^−2^). The starting biomass and composition of benthic aquatic invertebrates in the model were estimated from benthic rock sampling (n = 6), conducted in mid-August from each of the 32 study pools (H = 0.1±0.0039 g·m^−2^, G = 0.13±0.0052 g·m^−2^). The background rate of terrestrial prey inputs into Fox creek was estimated using pan traps (980 cm^2^) deployed for 24 hours approximately every ten days during the summer (July 1, July 9, July 19, August 1, August 12), at five locations across Fox creek. Pan traps were set above the stream surface with a few centimeters of water and 2–3 drops of surfactant to capture falling invertebrates. Emergence of aquatic invertebrates was quantified using replicate sticky traps (603 cm^2^) deployed perpendicular to the stream flow for 48 hours within 16 pools covered with enclosed plastic greenhouses, minimizing the likelihood of capturing invertebrates emerging from other stream reaches. All invertebrate data from sticky traps, benthic rock sampling, and pan traps were identified to family, and converted to biomass estimates using taxon specific length-weight relationships [39]–[41]. Starting values for the biomass of algae were estimated from unglazed ceramic tiles (23 cm^2^) incubated for 9 weeks in each of 32 study pools during peak summer productivity June 20^th^ - August 19^th^, 2010 (mean AFDM = 1.59±0.44 g·m^−2^). The background rate of terrestrial detritus input was estimated using a combination of leaf litter traps (0.25 m in diameter) that collected in-fall from the riparian forest, and lateral debris traps (0.5 long) deployed along the stream edge parallel to the bank to sample surface litter transport. Buckets and litter baskets were deployed continuously at 10 locations throughout Fox creek and collected every 2–4 weeks between early summer and early fall (Jul. 14, 2004–Aug. 10, 2004 & Jun. 21, 2005–Sept. 10, 2005). Samples were dried at 60°C for 48 hours and then weighed, and dry weights were converted to a flux of mass per unit wetted stream area per day. Daily litter subsidy was estimated as (1.7±1.5 g·m^−2^·day^−1^).

### Model Scenarios and Evaluation

Using the modeling framework described above, we evaluated how variation in the availability of terrestrial prey subsidies and the relative abundance of armored invertebrates influences the strength of top-down interactions in tributary stream food webs. Five levels of terrestrial prey subsidy fluxes were simulated, including 0%, 50%, 100%, 150% and 200% of background subsidy input rates. To test the degree to which prey vulnerability mediates the strength of top-down control, subsidies were held at reference conditions (0.261 g·m^−2^·day^−1^ prey, 1.7 g·m^−2^·day^−1^ detritus) and food web responses to factorial combinations of both salamanders and steelhead trout presence or absence were simulated across a range of aquatic invertebrate compositions, ranging from 0% to 100% armored for both functional groups of invertebrates. Each subsidy and vulnerability scenario was run for 90 daily time steps simulating a period of over-summer growth. At the conclusion of the 90 day model run, changes in the biomass of steelhead trout, salamanders, vulnerable invertebrates, armored invertebrates, algae, and the standing pool of terrestrial detritus were used to compare food web responses to subsidy scenarios. Light availability was constant across all model simulations, allowing us to specifically test the response of the model community to variation in prey subsidy magnitude and prey vulnerability.

The consequences of each model scenario were evaluated using log response ratios. Log response ratios (LRR) were calculated as,
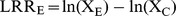
where E is the “experiment”, in our case simulations of different subsidy inputs, prey vulnerability, and predator assemblage, X_E_ is the biomass of a given trophic group at day 90 (e.g. steelhead trout, salamanders, armored and vulnerable invertebrates) in response to that change, and X_C_ is the biomass of that trophic group in the reference model (e.g. 100% prey). Log response ratios (LRR) offer a simple, easily interpretable measure for the interpretation relative magnitude of community level changes induced by each model scenario [42].

## Results

### Food Web Responses to Variation in Terrestrial Prey Subsidies

**Figure 1:**
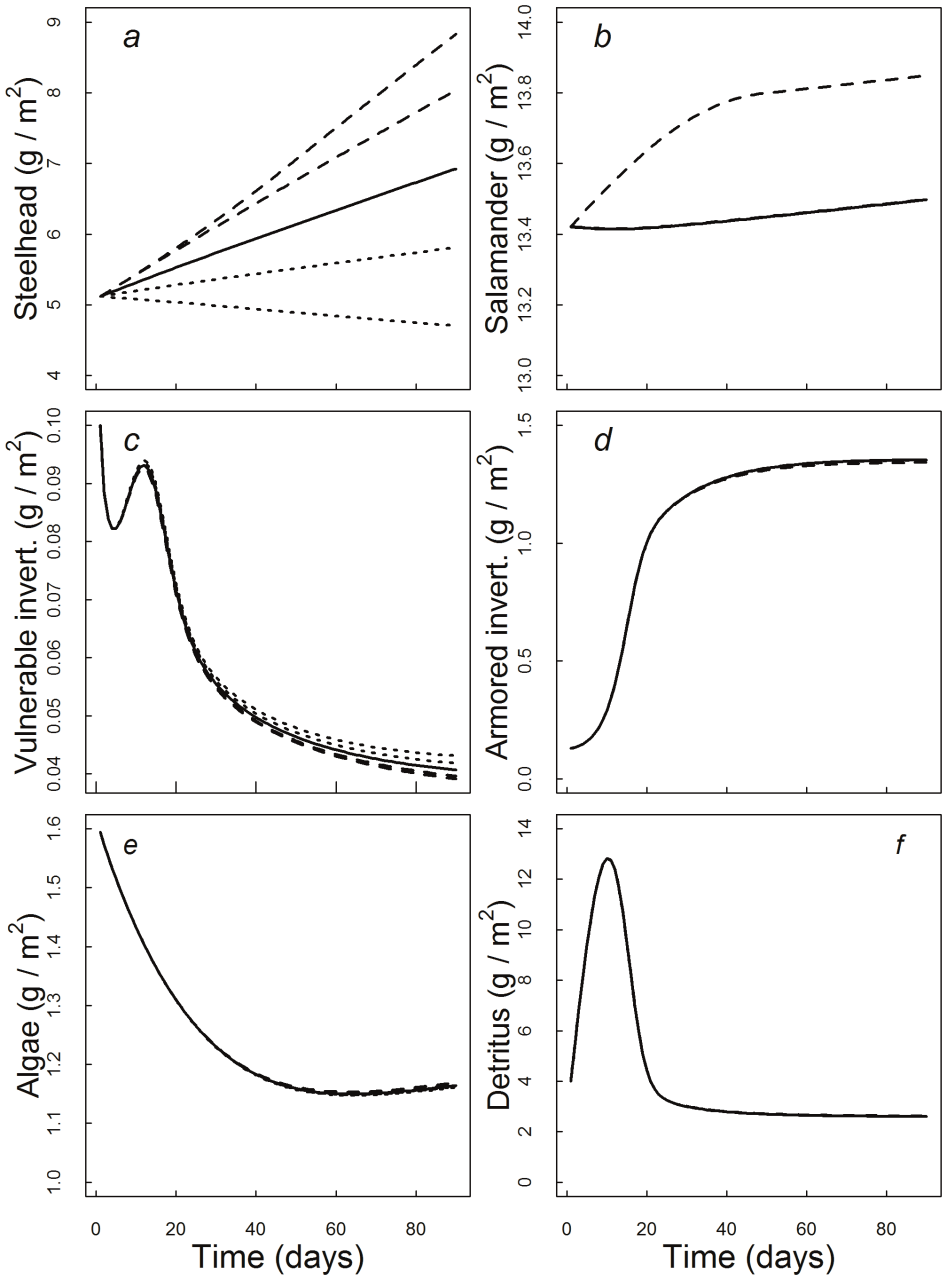
Response of model food web to changes in the magnitude of terrestrial prey subsidies in 90-day simulations for predators (top panels, a,b), primary consumers (middle panels, c,d), and basal resource pools (bottom panels, e,f). Solid line indicates natural background level of terrestrial prey inputs (100%, 0.261 g·m^−2^), short dashed lines represent reduced prey subsidies relative to natural (0%, 50%), and long dashed lines represent elevated prey subsidies relative to natural (150%, 200%).

As predicted, predator biomass increased with higher rates of terrestrial prey subsidy inputs, with an 88% increase in steelhead biomass (Fig. 1a) and a 2.6% increase in salamander biomass (Fig. 1b) across the range of input rates we considered (0–200% of background). However, the increased biomass of subsidized predators did not strengthen top-down control in the model food web, with almost no variation across subsidy scenarios in the final biomass of either vulnerable (Fig. 1c) or armored (Fig. 1d) primary consumers, or the final biomass of algae (Fig. 1e) or detritus (Fig. 1f). Both predator species in our model system consumed aquatic and terrestrial prey during the simulation, yet model results across the range of simulated subsidy inputs (0–200% of background) suggest that variation in predatory steelhead biomass is driven almost entirely by inputs of terrestrial prey (Fig. 1a). Increasing the magnitude of prey subsidies from 0% to 200% of background rates resulted in a sharp increase in steelhead biomass at the end of the 90-day model run (LRR_200%_ = 0.63) (Fig. 1a). In contrast to the patterns observed in steelhead biomass, salamanders benefitted minimally from terrestrial prey subsidies (Fig. 1b). Salamander biomass increased only 2.7% when prey subsidies increased from 0% to 200% of background rates (LRR_200%_ = 0.026).

Across model simulations of terrestrial prey subsidies, scenarios that included the presence of predatory salamanders and steelhead suggested that the biomass of vulnerable aquatic invertebrates was strongly limited by predators (LRR_trout_ = −3.34, LRR_salamander_ = −2.91). In both functional groups of invertebrates (detritivore and herbivore), predators induced a sharp decline of vulnerable aquatic invertebrates to a relatively low (0.041 g · m^−2^) but stable biomass (Fig. 1c). However, increases in terrestrial prey subsidies, and the concurrent increase in predator biomass, strengthened top-down control of vulnerable invertebrate biomass only modestly (LRR_200%_ = −0.097), with very little variation observed across terrestrial subsidy levels (Fig. 1c). In contrast, the biomass of armored aquatic invertebrates was positively affected by the presence of both predators (LRR_trout = _0.65, LRR_salamander = _0.2) and was not strongly influenced by the magnitude of the influx of terrestrial prey (LRR_100% = _0.0012, LRR_200%_ = −0.0078). By day 90 of the reference conditions model, armored aquatic invertebrates were more than an order of magnitude more abundant than vulnerable aquatic invertebrates, and comprised 97% of the total primary consumer biomass in our model food web (Figs 1c, 1d).

**Figure 2:**
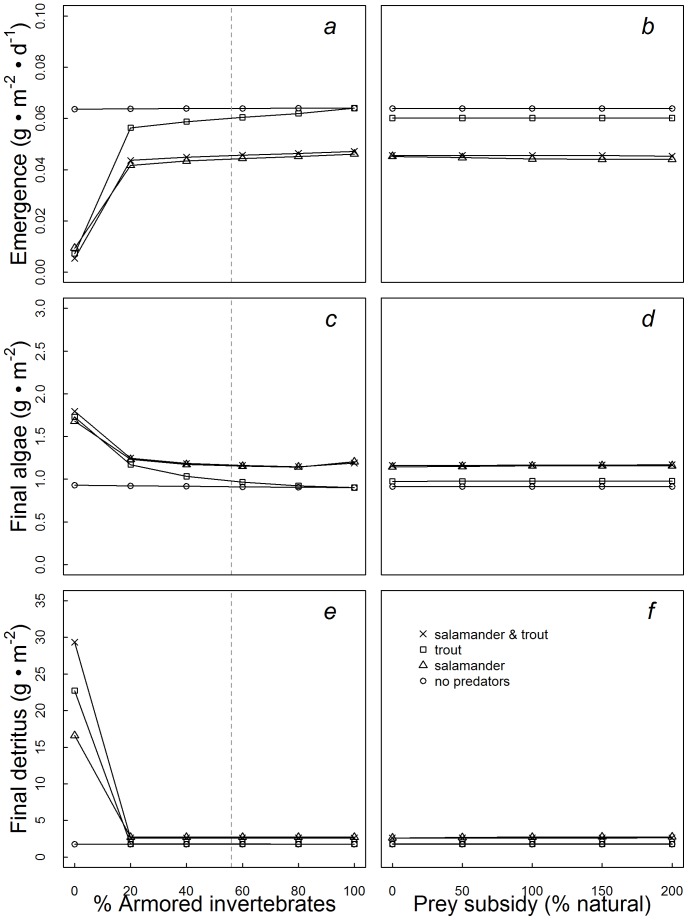
Biomass of emerging aquatic invertebrates (a,b), algae (c,d), and detritus (e,f) after 90-day model simulations, in the presence of steelhead trout (square), salamanders (triangle), both predators (cross), or no predators (circle). Each predator combination was modeled under six invertebrate assemblage scenarios (left panels), ranging from only vulnerable invertebrates (0% armored) to 100% armored, and also six levels of terrestrial prey influx rates (right panels, 0%, 50%, 100%, 150%, 200%). Reference conditions for invertebrate vulnerability (left panels, 56% armored) are represented by dashed vertical lines.

**Figure 3:**
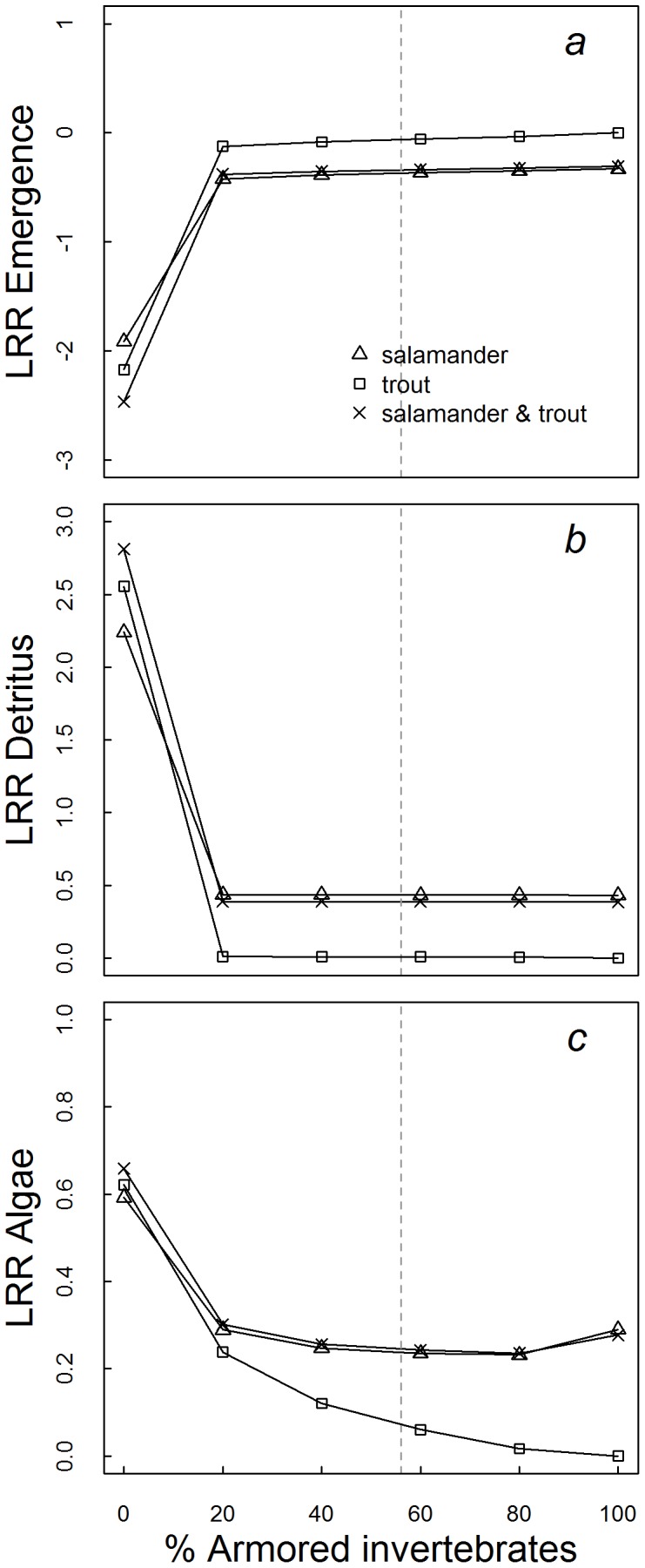
Strength of top-down control as indicated by log response ratios (LRR) of the biomass on day 90 of (a) emerging aquatic invertebrates, (b) detritus, and (c) algae to the presence of steelhead trout, salamanders, and both predators. Each predator combination was modeled under six different aquatic invertebrate assemblages ranging from only vulnerable invertebrates (0% armored) to 100% armored. LRR’s of each predator combination are expressed as a relative measure of the natural log of the final biomass (day 90) of each group in the presence minus the absence of the predator(s). Reference conditions for invertebrate vulnerability (56% armored) represented by dashed vertical lines.

For those aquatic invertebrates that escaped predation, the mean emergence rate of aquatic invertebrates into aerial adults over the course of the 90-day reference model simulation was estimated to be 0.045 g·m^−2^·day^−1^. Because armored invertebrates comprised the majority of the biomass of emerging aquatic invertebrates, variation in prey subsidies did not affect the magnitude of emergence rates (LRR_100%_ = −0.00022, LRR_200%_ = −0.0059) (Figs 2a, 3a). While variation in terrestrial subsidies did not affect emergence rates, under reference conditions the presence of predatory salamanders decreased emergence by 31%, while the presence of steelhead resulted in little change in daily emergence (5%) (Fig. 2b).

In general, increases in the availability of terrestrial subsidies increased predator biomass, but did not result in cascading trophic interactions to the level of the two basal energy sources (algae, detritus). Across the range of terrestrial prey subsidy scenarios, subsidies exerted little effect on *in situ* primary productivity (LRR_100%_ = 0.0026, LRR_200%_ = 0.0042) (Figs 1e, 2d). When subsidies were held at reference levels, the presence of trout had almost no effect on the final biomass of algae (LRR_trout_ = 0.069) but salamanders did increase primary productivity slightly (LRR_salamander = _0.24) (Figs. 2c, 3b). Similarly, terrestrial prey subsidies did not have strong effects on the pool of detrital biomass measured at the end of the 90-day simulation (LRR_100%_ ∼ 0, LRR_200%_ = 0.0084) (Figs 1f, 2f). Under reference conditions, predation by salamanders resulted in modest increases in detrital accumulation (LRR_salamander = _0.43), while the presence of steelhead did not change the final biomass of detritus (LRR_trout = _0.0086) (Figs 2e, 3c).

### Food Web Responses to Variation in Aquatic Prey Vulnerability

The presence of armored aquatic invertebrates in our model food webs limited top-down control and increased the biomass of emerging aquatic invertebrates. Across the range of invertebrate vulnerability to predation (0–100% armored), when 20% or more of the initial biomass of benthic invertebrates (herbivores and detritivores combined) were armored, strong top-down control by predators was absent (Figs 2 left panels, 3). Across all simulations, the final biomass of armored invertebrates was limited primarily by the availability of basal resources rather than the presence of predators. Salamanders were able to exploit armored prey up to a maximum of 6.5% of their daily consumption rates, which resulted in moderate top-down control with 27% higher algal biomass when salamanders were present (Fig. 2c). When all aquatic invertebrates in the food web were modeled as vulnerable to predation (0% armored), both salamanders and steelhead trout depressed the biomass of all aquatic invertebrates, triggering a strong trophic cascade that resulted in a 51% increase in algal biomass (LRR_trout = _0.62, LRR_salamander = _0.59 ) (Figs. 2c, 3b) and an 16-fold increase in the accumulation of detritus (LRR_trout = _2.55, LRR_salamander = _2.24 ) (Figs 2e, 3c). In the absence of armored invertebrates, predation by salamanders and trout similarly depressed the daily flux of emerging aquatic invertebrates into the riparian forest by 88% relative to the reference assemblage (LRR_trout_ = −2.17, LRR_salamander_ = −1.91). By contrast, in scenarios that included a relatively small proportion of armored invertebrates (20%) top-down control of insect emergence by salamanders and trout was weak or absent (LRR_trout_ = −0.12, LRR_salamander_ = −0.42). All told the total biomass of emerging invertebrates under reference conditions (56% armored) exceeded that consumed by salamanders and trout by 273%, routing most energy from the aquatic ecosystem into the riparian forest (Figs 2a, 3a). When armored invertebrates were not included in the model community (0% armored scenario), the balance between the invertebrate biomass consumed by aquatic predators and that emerging was reversed, with predation by salamanders and trout removing 1380% more biomass than what emerged from the stream.

## Discussion

The results of the modeled scenarios suggest that the effects of terrestrial prey subsidies on tributary food webs are limited to positive effects for top predators, and that prey subsidies do not elicit short term changes in the strength of top-down control at the level of primary consumers. Consistent with a large body of research documenting the benefits of prey subsidies for predators [8], [9], [11], prey inputs into the model community led to increased predator biomass, with nearly twice as much steelhead biomass when subsidies were 200% of background compared to when they were absent (Fig. 1a). Drift feeding steelhead frequently feed on the surface where terrestrial prey are first encountered, and terrestrial prey subsidies only benefitted the less mobile, more benthic giant salamanders when steelhead were unable to exploit all of the terrestrial prey. When fluxes of terrestrial prey were simulated at twice the empirically observed rate (200%) salamander biomass increased modestly, as large inputs of terrestrial prey exceeded the consumptive capacity of steelhead trout (ie. X>c_t_T) (Fig. 1b). However, contrary to theory [2], [10], [13], the observed short-term increases in the biomass of either predator did not produce cascading trophic interactions in our model food web (Fig. 2, right panels).

The impact of predation by steelhead and salamanders on the biomass of aquatic invertebrates depended acutely on the degree of prey vulnerability. The community level responses to predation as the proportion of armored invertebrates in the benthic community increased indicate that the presence of predator-invulnerable primary consumers dramatically reduced the ability of predators to exert top-down control, even under the highest levels of terrestrial subsidy (200% background). Changes in the vulnerability of the aquatic consumer assemblage resulted in greater changes in trophic level biomass and stronger top-down control than did variation in prey subsidy magnitude over the short term, suggesting that the vulnerability of *in situ* prey may dictate the degree to which resource subsidies propagate through recipient food webs. Vulnerable aquatic invertebrates, including both herbivores and detritivores, were reduced to low levels (0.041 g· m^−2^) over the course of the 90-day simulations by both predator species regardless of the availability of terrestrial subsidies (Fig. 1c). This pattern suggests that in-stream predators are capable of effectively exploiting nearly all vulnerable prey regardless of overall resource availability. These model results match a recent experimental test of the role of predation in the same tributary stream, both *D. tenebrosus* and *O. mykiss* exerted top-down control of predator vulnerable aquatic invertebrates during peak summer productivity [35]. By contrast, armored aquatic invertebrates (e.g. Order Trichoptera and Coleoptera) actually benefitted slightly from the presence of trout and salamanders as predation on vulnerable aquatic taxa reduced competition between armored invertebrates (herbivores and detritivores) and their vulnerable counterparts (Fig. 1d). Thus, when the aquatic invertebrate assemblage consists of predator invulnerable taxa, secondary production may be limited by resource availability [15], [18], and abiotic factors such as light and nutrient availability may play a much larger role in determining secondary productivity [43], [44].

The presence of armored taxa has been found to limit the ability of predators to induce trophic cascades [15], [17], and we propose that the absence of a strong cascading response to increased terrestrial prey subsidies in the model food web was attributable to the high abundance (∼60% of biomass) of armored invertebrates. In model scenarios that included only vulnerable invertebrates (0% armored), the high biomass of subsidized predators relative to aquatic prey led to strong top-down control and trophic cascades, matching theoretical predictions [2], [10], [13]. However, when 20% or more of the initial aquatic invertebrate biomass was modeled as armored, the model community dynamics underwent a rapid change away from predator control (Fig. 3). Salamanders were capable of exploiting armored invertebrates on a limited basis, and as such, did exert some control on lower trophic levels across the range of benthic community vulnerability scenarios we examined (Fig. 3). However, the top down effects of salamanders were small relative to those produced when the abundance of armored aquatic invertebrates was less than 20% (Fig. 2, left panels).

The observation that prey vulnerability can mediate the strength of predation within food webs is consistent with findings from a range of ecosystems and food web types [15], [16], [18], [19]. Predator induced defenses in zooplankton communities have been shown to dramatically reduce predation rates preventing trophic cascades [21], and large body size of primary consumers can limit vulnerability to predation in terrestrial ecosystems [18]. Invulnerable primary consumers can decouple primary consumer dynamics from higher trophic levels acting as “trophic cul-de-sacs” which limit the flow of energy through the food web, and depress the biomass and diversity of other consumers via competition [19]. In stream food webs, armored caddisfly larvae are largely unavailable to a wide range of predators [15], [20], [25], [35]. They are often the most abundant primary consumers in tributary streams, limiting the availability of algal energy to competitors as well as higher trophic levels [20], arresting trophic cascades [15], [17]. In the model stream food web armored invertebrates appear to reduce the productivity of aquatic food webs for higher order consumers and limit the scope for top-down control, shifting the balance of food web regulation from top-down control to resource limitation.

Despite their advantages, the morphological adaptations of armored aquatic invertebrates that act to reduce their vulnerability to predation likely come at a cost. Many aquatic armored taxa are hypothesized to have reduced mobility, which can limit dispersal abilities, their ability to track resources in space and time, and their ability to avoid disturbance. Wootton and colleagues [15] found that the strength of trophic cascades in a river ecosystem could be predicted by the intensity of scouring winter floods, which induce disproportionately high mortality among heavily armored grazers, leaving an invertebrate community dominated by more predator-vulnerable taxa which are readily exploited by predatory fish. Dispersal is an important driver of the distribution patterns of aquatic invertebrates [45], and flood disturbance may serve to maintain the diversity of the aquatic invertebrate community if non-armored invertebrates have higher survival during floods or if they more readily colonize stream reaches following scouring floods [43]. Morphological and behavioral traits that offer defense against predators can also come at the expense of growth rates and competitive ability [46], and prey species diversity is likely maintained in part by variation in the way that different species balance predation risk, foraging, and growth efficiency trade-offs [47]. However, many of these tradeoffs may manifest over longer time periods than a single growing season, and our model sought explicitly to address the implications of armoring for trophic interactions across shorter timescales. Future work should further explore the physiological and demographic consequences of prey armoring and how these tradeoffs ultimately influence the composition of stream invertebrate communities.

We found that the degree of prey armoring among aquatic invertebrate assemblages was a primary control on the magnitude of the reciprocal flux of aquatic prey into the terrestrial ecosystem. The high abundance (56%) of armored aquatic invertebrates in our reference model food web meant that very little of the energy entering the aquatic food web as terrestrial detritus was available to aquatic predators. Consequently, the majority of detrital energy that entered the aquatic food web was routed back into the terrestrial environment through the emergence of armored taxa as winged adults. Many riparian predators depend on emerging aquatic insects as prey [3], [9], [11] and our model scenarios suggest that the balance between terrestrial inputs (detritus) and emerging aquatic invertebrates can change dramatically in response to changes in the abundance of armored prey (Fig. 2a). The total flux of aquatic invertebrates back to the terrestrial ecosystem in our reference model (0.045 g·m^−2^·day^−1^) exceeded consumption of by local aquatic predators by 2.7 fold. This may represent an underestimate of predation rates, since we did not specifically account for predation occurring during emergence. The emergence process from the aquatic larval stage into aerial adult is known to subject aquatic insects to high predation risk, and aquatic predators may reduce the magnitude of aquatic emergence without inducing detectable top-down effects upon benthic invertebrate biomass [48]. However, in food web simulations that included only vulnerable invertebrates (0% armored), salamanders and trout consumption was almost 14 times greater than emergence, and the biomass of aquatic invertebrates emerging from the model stream food web was depressed roughly 8-fold (0.0054 g·m^−2^·day^−1^).

Our model was parameterized with empirically derived values from Fox Creek, a tributary of the South Fork Eel in the northern California Coast Range. We estimated summertime prey subsidy inputs of (0.261±0.051 g·m^−2^ ·day^−1^), however we should note that magnitude terrestrial prey subsidies to Fox Creek are at least an order of magnitude greater than those reported in other studies [7], [49], [54], [55]. Despite the variation in reported rates of terrestrial invertebrate inputs, our results are consistent with a large body of literature demonstrating the importance of prey subsidies for aquatic predators.

The use of a multi-trophic model facilitated exploration of the potential role of terrestrial prey subsidies in recipient food webs and simulated the interactions between food web members across a range of biologically plausible scenarios. Predators, prey, and basal energy were modeled as biomass pools allowing us to ask broad questions about the flow of energy across trophic levels and the interaction between prey subsidies and the composition of the prey community in mediating patterns of top-down regulation over short time scales. While these findings yield insight into the general dynamics of subsidized ecosystems, modeling complex ecological interactions necessarily involves simplifications, with a resulting loss in biological realism. For instance, prey selectivity by predators can lead to unforeseen effects of changes in prey availability in real food webs, and top down effects of stream predators may be reduced if they preferentially feed on allochtonous prey [5], [7]. However, steelhead and giant salamanders cannot necessarily increase exploitation of aquatic prey when terrestrial prey subsidies are eliminated over short time scales [35], [49]. Additionally, interactions between competing species can be size-structured rather than species-based [50], and may change throughout ontogeny [51]. Salamanders and trout may act as either predator and prey for one another [52], [53]. However, experimental evidence suggests that growth by salamanders and trout is not negatively affected by the presence of the other species [35], and predatory interactions between salamanders and trout were not included in the model. The timescale of our model simulations allowed us to ask questions about the short-term response of tributary stream food webs under each scenario without the added complexity of incorporating longer-term population level processes such as reproduction or movement. Over longer timescales, higher levels of terrestrial subsidies, if consistent, should result in higher abundance of top predators. However, this model explored dynamics during the short period of over-summer growth during the peak of annual productivity in streams and future studies should explore the longer term consequences of prey armoring and resource subsidies for stream community dynamics.

Overall, there was strong support for the hypothesis that stream predators benefit from terrestrial prey subsidies, with trout experiencing higher (∼90%) summer growth in the presence of prey subsidies. However, the abundance of armored primary consumers in the aquatic invertebrate assemblage had a much larger effect on the strength of food web interactions than changes in subsidy magnitude. These results lead to the observation that increases in the biomass of subsidized predators do not necessarily translate into changes in the dynamics of recipient food webs, especially when prey invulnerability arrests the propagation of subsidies through food webs. Increasing the abundance of armored invertebrates in the model food web resulted in a dramatic shift in food web regulation, with top-down control decreasing markedly when armored consumers comprised 20% or more of the invertebrate biomass. The empirically derived starting conditions and parameter values suggest that the absence of strong cascading interactions observed in tributary streams [17], [35], [49] may be common if the data are representative of other tributary systems, especially the relatively high biomass (∼60%) of armored invertebrates. Overall, we find that morphological adaptations of prey that limit their susceptibility to predation appear to play a key role in the persistence of prey assemblages, and likely play an important and underappreciated role in mediating the response of food webs to resource subsidies. Such adaptations may be ubiquitous among prey in highly subsidized ecosystems where predator biomass greatly exceeds the capacity of the local food web (e.g. *in situ* resources). We conclude that a deeper understanding of patterns of prey vulnerability across different subsidized ecosystems would represent an important advance in understanding the dynamics of spatially subsidized food webs.

## References

[1] Vannote RL, Minshall GW, Cummins KW, Sedell JR, Cushing CE (1980) The river continuum concept. Canadian Journal of Fisheries and Aquatic Science 37: 130–137.

[2] Polis GA, Anderson WB, Holt RD (1997) Toward an integration of landscape and food web ecology: the dynamics of spatially subsidized food webs. Annual Review of Ecology, Evolution, and Systematics 28: 289–316.

[3] Nakano S, Murakami M (2001) Reciprocal subsidies: dynamic interdependence between terrestrial and aquatic food webs. PNAS 98: 166–170. 11136253 10.1073/pnas.98.1.166 PMC14562

[4] Marczak LB, Thompson RN, Richardson JS (2007) Meta-analysis: trophic level, habitat, and productivity shape the food web effects of resource subsidies. Ecology 88: 140–148. 17489462 10.1890/0012-9658(2007)88[140:mtlhap]2.0.co;2

[5] Nakano S, Miyasaka H, Kuhara N (1999) Terrestrial-aquatic linkages: riparian arthropod inputs alter trophic cascades in a stream food web. Ecology 80: 2435–2441.

[6] Murakami M, Nakano S (2002) Indirect effect of aquatic insect emergence on a terrestrial insect population through predation by birds. Ecology Letters 5: 333–337.

[7] Baxter CV, Fausch KD, Murakami M, Chapman PL (2004) Fish invasion restructures stream and forest food webs by interrupting reciprocal prey subsidies. Ecology 85: 2656–2663.

[8] Polis GA, Hurd SD (1995) Extraordinarily high spider densities on islands: flow of energy from the marine to terrestrial food webs and the absence of predation. PNAS 92: 4382–4386. 7753815 10.1073/pnas.92.10.4382 PMC41948

[9] Sabo JL, Power ME (2002) River-watershed exchange: effects of riverine subsidies on riparian lizards and their terrestrial prey. Ecology 83: 1860–1869.

[10] Holt RD (1984) Spatial heterogeneity, indirect interactions, and the coexistence of prey species. American Naturalist 124: 377–406. 10.1086/284280 29519131

[11] Henschel JR, Mahsberg D, Stumpf H (2001) Allochthonous aquatic insects increase predation and decrease herbivory in river shore food webs. Oikos 93: 429–438.

[12] Polis GA, Hurd SD (1996) Allochthonous inputs across habitats, subsidized consumers, and apparent trophic cascades: examples from the ocean-land interface. In: Polis GA, Winemiller KO, editors. Food Webs: Integration of Patterns and Dynamics. London: Chapman & Hall. 275–285.

[13] Leroux SJ, Loreau M (2008) Subsidy hypothesis and strength of trophic cascades across ecosystems. Ecology Letters 11: 1147–1156. 18713270 10.1111/j.1461-0248.2008.01235.x

[14] McCann K, Hastings A, Huxel GR (1998) Weak trophic interactions and the balance of nature. Nature 395: 794–798.

[15] Wootton JT, Parker MS, Power ME (1996) Effects of disturbance on river food webs. Science 273: 1558–1561.

[16] Chase JM (1999) Food web effects of prey size refugia: variable interactions and alternative stable equilibria. American Naturalist 154: 559–570. 10.1086/303260 10561128

[17] Ruetz CR, Newman RM, Vondracek B (2002) Top-down control in a detritus-based food web: fish, shredders, and leaf breakdown. Oecologia 132: 307–315. 28547366 10.1007/s00442-002-0953-1

[18] Sinclair ARE, Mduma S, Brashares JS (2003) Patterns of predation in a diverse predator-prey system. Nature 425: 288–290. 13679915 10.1038/nature01934

[19] Bishop MJ, Kelaher BP, Alquezar R, York PH, Ralph PJ, et al (2007) Trophic cul de-sac, *Pyrazus ebeninus*, limit trophic transfer through an estuarine detritus-based food web. Oikos 116: 427–438.

[20] McNeely C, Finlay JC, Power ME (2007) Grazer traits, competition, and carbon sources to a headwater-stream food web. Ecology 88: 391–401. 17479757 10.1890/0012-9658(2007)88[391:gtcacs]2.0.co;2

[21] van der Stap I, Vos M, Verschoor AM, Helmsing NR, Mooij WM (2007) Induced defenses in herbivores and plants differentially modulate a trophic cascade. Ecology 88: 2474–2481. 18027750 10.1890/07-1731.1

[22] Wallace JB, Eggert SL, Meyer JL, Webster JR (1997) Multiple trophic levels of a forest stream linked to terrestrial litter inputs. Science 277: 102–104.

[23] Gutierrez AP, Baumgaertner JU, Summers CG (1984) Multi-trophic models of predator-prey energetic: I. age specific energetic models-pea aphid *Acyrthosiphon pisum* (homoptera: aphidae) as an example. Canadian Entomologist 116: 923–932.

[24] Finlay JC (2001) Stable-carbon-isotope ratios of river biota: implications for energy flow in lotic food webs. Ecology 82: 1052–1064.

[25] Parker MS (1994) Feeding ecology of stream-dwelling pacific giant salamander larvae (*Dicamptodon tenebrosus*). Copeia 1994, 705–718.

[26] Limm MP, Power ME (2011) The caddisfly *Dicosmoecus gilvipes*: making a case for a functional role. Journal of the North American Benthological Society 30: 485–492.

[27] Johnson MG, Brinkhurst RO (1971) Production of benthic macroinvertebrates of Bay of Quinte and Lake Ontario. Journal of the Fisheries Research Board of Canada 28: 1699–1714.

[28] McDiffett WF (1970) The transformation of energy by a stream detritivore, Pteronarcys Scotti (Plecoptera). Ecology 51: 976–988.

[29] Grafius E, Anderson NH (1979) Population dynamics, bioenergetics, and the role of Lepidostoma Quercina Ross (Trichoptera: Lepidostomatidae) in an Oregon woodland stream. Ecology 60: 433–441.

[30] Benke AC, Wallace JB (1980) Trophic basis of production among net-spinning caddisflies in a southern Appalachian stream. Ecology 61: 108–118.

[31] Holling CS (1959) Some characteristics of simple types of predation and parasitism. Canadian Entomologist 91: 385–398.

[32] Ware DM (1972) Predation by rainbow trout (*Salmo gairdneri*): the influence of hunger, prey density, and prey size. Journal of the Fisheries Research Board of Canada 29: 1193–1201.

[33] Crowder LB, Cooper WE (1982) Habitat structural complexity and the interaction between bluegills and their prey. Ecology 63: 1802–1813.

[34] Galbraith Jr MG (1967) Size-selective predation on daphnia by rainbow trout and yellow perch. Transactions of the American Fisheries Society 96: 1–10.

[35] Atlas WA, Palen WJ, Courcelles DM, Munshaw RG, Monteith ZL (2013) Dependence of stream predators on terrestrial prey fluxes: food web responses to subsidized predation. Ecosphere 4.

[36] Johnson JH, Ringler NH (1980) Diets of juvenile coho salmon (*Oncorhynchus kisutch*) and steelhead trout (*Salmo gairdneri*) relative to prey availability. Canadian Journal Zoology 58: 553–558.

[37] Pauly D, Christensen V (1995) Primary production required to sustain global fisheries. Nature 374: 255–257.

[38] Wurtsbaugh WA, Davis GE (1977) Effects of temperature and ration level on the growth and food conversion efficiency of *Salmo gairdneri*, Richardson. Journal of Fish Biology 11: 87–98.

[39] Hodar JA (1996) The use of regression equations for estimation of arthropod biomass in ecological studies. Acta Oecologia 17: 421–433.

[40] Benke AC, Huryn AD, Smock LA, Wallace JB (1999) Length-mass relationships for freshwater macroinvertebrates in North America with particular reference to the southeastern United States. Journal of the North American Benthological Society 18: 308–343.

[41] Sabo JL, Bastow JL, Power ME (2002) Length-mass relationships for adult aquatic and terrestrial invertebrates in a California Watershed. Journal of the North American Benthological Society 21: 336–343.

[42] Hedges LV, Gurevitch J, Curtis PS (1999) The meta-analysis of response ratios in experimental ecology. Ecology 80: 1150–1156.

[43] Power ME, Dietrich WE (2002) Food webs in river networks. Ecological Research 17: 451–471.

[44] Schade JD, MacNeill K, Thomas SA, McNeely FC, Welter JR, et al (2011) The stoichiometry of nitrogen and phosphorus spiraling in heterotrophic and autotrophic streams. Freshwater Biology 56: 424–436.

[45] Malmqvist B (2002) Aquatic invertebrates in riverine landscapes. Freshwater Biology 47: 679–694.

[46] Wellborn GA (2002) Trade-off between competitive ability and antipredator adaptation in a freshwater amphipod species complex. Ecology 83: 129–136.

[47] McPeek MA, Grace M, Richardson JML (2001) Physiological and behavioral responses to predators shape the growth/predation risk trade-off in damselflies. Ecology 82: 1535–1545.

[48] Wesner JS (2012) Predator diversity effects cascade across an ecosystem boundary. Oikos 121: 53–60.

[49] Zhang Y, Richardson JS (2011) Contrasting effects of cross-ecosystem subsidies and predation on benthic invertebrates in two Pacific coastal streams. Aquatic Sciences 73: 53–62.

[50] Jennings S, Greenstreet SPR, Hill L, Piet GJ, Pinnegar JK, et al (2002) Long-term trends in the trophic structure of the North Sea fish community: evidence from stable-isotope analysis, size-spectra and community metrics. Marine Biology 141: 1085–1097.

[51] Werner EE, Gilliam JF (1984) The ontogenic niche and species interactions in size-structured populations. Annual Review of Ecology, Evolution, and Systematics 15: 393–425.

[52] Parker MS (1993) Predation by Pacific giant salamander larvae on juvenile steelhead trout. Northwestern Naturalist 74: 77–81.

[53] Resetarits WJ (1991) Competitive asymmetry and coexistence in size-structured populations of brook trout and spring salamanders. Oikos 73: 188–198.

[54] Kawaguchi Y, Nakano S (2001) Contribution of terrestrial invertebrates to the annual resource budget for salmonids in forest and grassland reaches of a headwater stream. Freshwater Biology 46: 303–316.

[55] Eberle LC, Stanford JA (2010) Importance and seasonal availability of terrestrial invertebrates as prey for juvenile salmonids in floodplain spring brooks of the Kol River (Kamchatka, Russian Federation). River Research and Applications 26: 682–694.

